# Segmentation of Retinal Blood Vessels Based on Cake Filter

**DOI:** 10.1155/2015/137024

**Published:** 2015-11-09

**Authors:** Xi-Rong Bao, Xin Ge, Li-Huang She, Shi Zhang

**Affiliations:** School of Information Science and Engineering Northeastern University, Shenyang, Liaoning 110819, China

## Abstract

Segmentation of retinal blood vessels is significant to diagnosis and evaluation of ocular diseases like glaucoma and systemic diseases such as diabetes and hypertension. The retinal blood vessel segmentation for small and low contrast vessels is still a challenging problem. To solve this problem, a new method based on cake filter is proposed. Firstly, a quadrature filter band called cake filter band is made up in Fourier field. Then the real component fusion is used to separate the blood vessel from the background. Finally, the blood vessel network is got by a self-adaption threshold. The experiments implemented on the STARE database indicate that the new method has a better performance than the traditional ones on the small vessels extraction, average accuracy rate, and true and false positive rate.

## 1. Introduction

The retinal vasculature is the only component of the body's circulatory system that can be observed noninvasively by optical means. A large variety of diseases such as hypertension and diabetes affect the vasculature in a way that may cause geometrical and functional retinal vasculature changes. Therefore, the retinal image can be used in the diagnosis of not only glaucoma, macular degeneration, and other eye diseases but also diabetes mellitus, atherosclerosis, and other cardiovascular diseases. This makes the retinal vasculature have an important value on clinical medicine [1–3]. The extraction of retinal image network of blood vessels becomes a difficult problem in the retinal image processing and analysis [4–6] for the following reasons: (1) the retinal blood vessel is full of small blood vessels; (2) the network of blood vessels is complex; and (3) the illumination is uneven when acquiring images leading to the fact that the retinal image contrast is low.

The existing algorithms of retinal blood vessels segmentation can be divided into two classes [1, 2]: the pixel classification [7, 8] and the vessel tracking. Among the methods of pixel classification, Chaudhuri proposed a method using a Gaussian rotating matched filter (MFR) for segmentation of the retinal [9], which showed a good performance of main vessels and a lack of minutiae. Hoover et al. improved the MFR image using local threshold probing technique and region-based attributes of the pixels to extract the network of vessels [10]. However, its computation is very complex. Soares et al. used Gabor wavelet to extract areas with standard image characteristics as the segmentation results [11], which used a number of standard images segmented in advance and in most cases it cannot be implemented. Yao and Chen described a segmentation approach based on transition region using optimal entropy to extract the main vessels and two-dimensional Otsu to complete the extraction of transition region [12]. Its amount of calculation is large.

The other method is vessel tracking. This method extracts vascular network using the principle of recursive growth and it is greatly influenced by the initial point selection. Sun describes an adaptive tracking algorithm based on the characteristics of vessels as center-line, direction, and diameter [13]. Fraz et al. introduced an edge and center-line tracking algorithm based on direction field of retinal blood vessels [2]. Chen presented a vessel tracing method based on Hessian matrix [14]. Compared to pixel classification, this kind of method can segment the width of blood vessels accurately. However, this kind of method is not consistent with the real time requirements of the algorithm.

In view of the good directivity in local retinal vessels and low contrast image, this paper proposes a retinal blood vessels segmentation algorithm based on cake filter. Compared with the above segmentation methods, the cake filter has strong directivity, which can detect the slender structure of the image efficiently. It also is orthogonality, which can save more details and reduce the redundancy of the image, so that it will improve the accuracy of segmentation.

This paper first introduces the structure and principle of cake filter and the verification of its properties in Section 2. Then the segmentation algorithm based on cake filter is introduced in Section 3, followed by the test of the algorithm using STARE fundus image library and the analysis of comparing with classical algorithms such as MFR in Section 4. Finally the paper concluded in Section 5.

## 2. The Structure of Cake Filter

### 2.1. Principle and Design of Cake Filter

Many of retinal vessels are bending and disperse in all directions. In order to get efficient segmentation of vessels in different directions, it requires a filtering in more than one direction, which needs good directivity of the filter. At the same time, in order to make the result clearer with no redundant, filter should be orthogonal [15]. In this paper, we call it a suitable filter which satisfied the above two conditions [16].

To meet the two characteristics of the filter, this paper designs a filter by using the following method, named “cake filter.” The cake filter *ψ* is symmetrical in *x*-axis and orthogonal in *y*-axis, which is

(1) $$\begin{array}{c} \psi \left(\;\vec{x}\;\right)=\psi_{\text{even}}\left(\;\vec{x}\;\right)-i\psi_{\text{odd}}\left(\;\vec{x}\;\right), \end{array}$$

where $\vec{x}=(x,y)$ is a set of vectors, which means two-dimensional coordinates of the filter. In the *ψ*_odd_(*x*, *y*) = *ℋ*[*ψ*_even_(*x*, ·)](*y*), *ℋ* is a Hilbert transformation.

For the convenience of calculation, we design a filter in Fourier field by dividing Fourier field into *N* equal parts and making each component represent a direction cake. The expression of time domain is as follows:

(2) $$\begin{array}{c} \psi^{\text{cake}}\left(\;x\;\right)=F^{-1}\left[\;{\tilde{\psi }}^{\text{cake}}\left(\;\omega \;\right)\;\right]G_{\sigma_{s}}\left(\;x\;\right). \end{array}$$

It is defined as a product of the inverse Fourier transform of the frequency domain function ${\tilde{\psi }}^{\text{cake}}(\omega )$ and a Gaussian filter, where *ℱ* ^−1^ means the two-dimensional inverse Fourier transform of ${\tilde{\psi }}^{\text{cake}}(\omega )$ and *G*_*σ*_*s*__ is a Gaussian filter, with a condition of 0 < 1 ≪ *σ*_*s*_, which prevents a long tail in time domain of the cake filter. The actual formulas of ${\tilde{\psi }}^{\text{cake}}$ are

(3) $$\begin{array}{c} {\tilde{\psi }}^{\text{cake}}\left(\;\rho ,\varphi \;\right)=B_{k}\left(\;\frac{\left(\varphi \mathrm{mod}2\pi \right)-\pi /2}{s_{\theta }}\;\right)M_{N}\left(\;\rho \;\right), \end{array}$$

where (*ρ*, *φ*) is a set of polar coordinates and *ω* = (*ρ* cos *φ*, *ρ* sin *φ*) in Fourier domain; *s*_*θ*_ = 2*πN* ^−1^ is the resolution of angular coordinate. *ℳ*_*t*_ is a radioactive function in Fourier domain; *B*_*k*_ is a K-B spline function. *ℳ*_*N*_ is expressed as follows:

(4) Mtρ=Gtρ∑j=0qddρ′Gtρ′ρ′=0ρjj!−1,Gtρ=12πte−ρ2/4t.

**Figure 1:**
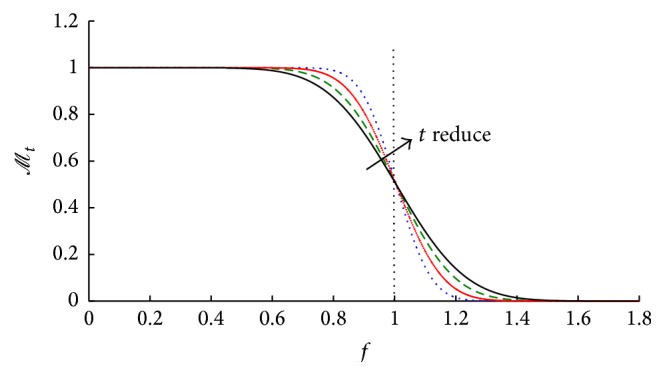
Value of function *ℳ*_*t*_(*ρ*).

The function *ℳ*_*t*_(*ρ*) is defined as a Gaussian function divided by its Taylor series expansion, where *j* = 0,1, 2,…, *q* is the order of the Taylor series expansion of *G*_*t*_(*ρ*), which ensures that the function *ℳ*_*t*_(*ρ*) will be changed slowly in the frequency range and changed rapidly in the edge of image. Mathematically, *ℳ*_*t*_(*ρ*) changes slowly in the range of *ρ* ∈ [0, *ϕ*] and changes rapidly in the edge as the function value switches from 1 to 0, which has been shown in Figure 1.

In Figure 1, the horizontal axis means frequency of image with *f* = 1 expressing the boundary of image. A set of curves in Figure 1 shows the variation when scale parameter *t* changes. When *t* reduces in the direction of the arrow, *ℳ*_*t*_(*ρ*) decreases faster in the range of *f* ∈ [0,1] in the edge of image, which means more frequency has been reserved. In other words, this property of *ℳ*_*t*_ makes the filter keep more details.

*B*_*k*_ can be represented as follows:

(5) Bkx=Bk−1∗B0x,B0x=1,if  −12<x<12,0,otherwise.

Table 1 shows the unilateral filter template corresponding to the different *N* value. In Table 1, the first three figures show the results of the cake filter and the last one is the result of Gabor filter. From these figures, it can be seen that, with a bigger value, the corresponding cake filter has a stronger directivity, but at the same time the calculation is more complex.

### 2.2. The Properties Verification of the Filter

As we know, a suitable filter should have a good directivity and orthogonality. The verification is carried out as follows.

#### 2.2.1. Directional Validation

Due to the directional characteristics of retinal vascular images, cake filter is needed to be used for directional test in different direction of retinal vessels.

Such an orientation score is obtained by the cake filter, labeled as *U*_*f*_:

(6) $$\begin{array}{c} U_{f}\left(\;x,\theta \;\right)=\left(\;\psi_{\theta }*f\;\right)\left(\;x\;\right),\;\psi_{\theta }\in L_{2}\left(R^{2}\right), \end{array}$$

where *ψ*_*θ*_ means a filter template in the *θ* direction, *U*_*f*_(*x*, *θ*) is the orientation, and *f* is a 2D digital image. Notice that *ψ*_*θ*_ ∈ *𝕃*_2_(*ℝ* ^2^) is the process of image processing in 2D.

**Figure 2:**
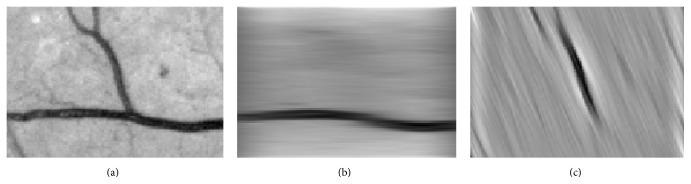
Orientation score based on cake filter.

**Figure 3:**
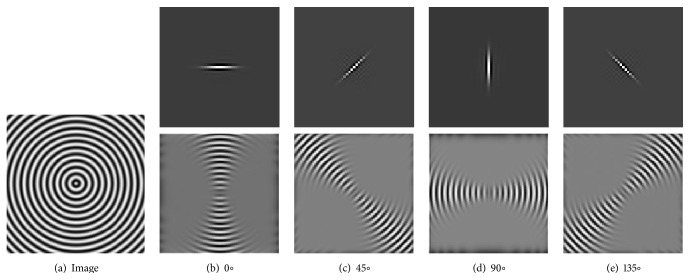
Orientation scores under different orientations.

Figures 2 and 3 show the orientation scores in different directions using cake filter.  Figure 2(a) presents the original image. Figures 2(b) and 2(c) show the real component of orientation scores under the horizontal and diagonal direction, respectively. It can be seen that the orientation score keeps a better result of vascular information in the same direction and at the same time filters the vessels information and noise of other directions, which means a good directivity.

#### 2.2.2. Orthogonal Validation

The orthogonality is an important component of the performance of a filter, which can remove the redundancy of the image well and guarantee the effect of image processing.

**Figure 4:**
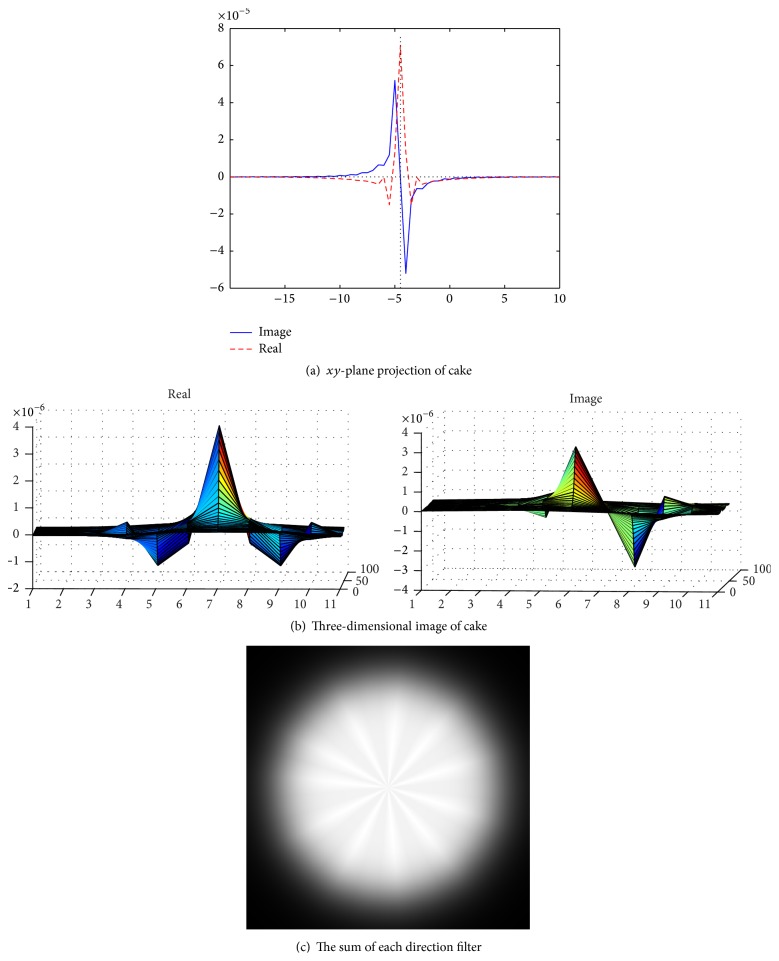
Quadrature property of the cake filter.

Figure 4 shows the validation of orthogonality of the cake filter.  Figure 4(a) is the projection of real component and imaginary component in the plane, and Figure 4(b) is the real component and imaginary component of 3D image. Obviously, the cake filter is symmetric in *x*-axis and matches formula (1), which means the filter is orthogonal.

As the cake filter is orthogonal, therefore, it should just cover all the frequency of Fourier domain with nonoverlapping theoretically, which proves that it is orthogonality again.

### 2.3. The Comparison of Cake and Gabor Filter

Gabor filter theory was first put forward by the British physicist Gabor [17]. Gabor filter is directional and consistent with the receiving field model of mammalian retinal nerve cells. Therefore, there are many scholars that propose retinal blood vessels segmentation algorithms based on Gabor filter [18–20].

We can see from Table 1, though Gabor filter has directivity, the cake filter has a better performance. Besides, Gabor filter consists of a set of nonorthogonal bases (as shown in Table 1), which leads to a redundancy image after filtering and makes a negative result of segmentation. And there is no such problem in cake filter due to its orthogonality.

**Figure 5:**
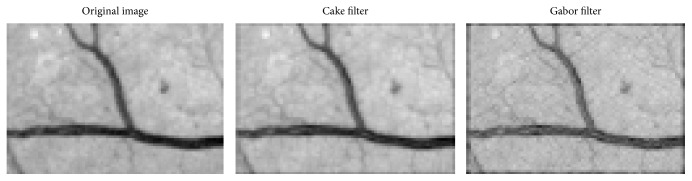
Comparison of cake and Gabor filter.

Figure 5 shows the fusion of the real component from the result of cake filter and Gabor filter; what is obvious is that the cake filter is basically the same as the original image, keeping more information of image, but the Gabor filter is not. It shows that the cake filter has good directivity to be applied to the vessel extraction comparing with the Gabor filter.

## 3. Retinal Blood Vessels Segmentation Algorithm Based on the Cake Filter

**Figure 6:**
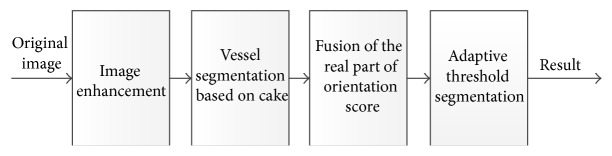
Process of cake filter method.

From the above discussion, we can conclude that the cake filter has strong directionality, which can detect the linear structure of images. And retinal blood vessels have a linear structure and certain direction in local areas with noise distributed in mess; therefore, it can be used to distinguish the vessels and noise, so as to extract the retinal vascular network. The steps of the proposed algorithm are shown in Figure 6, which includes the image enhancement, the vessels segmentation, the real component fusion, and the threshold segmentation. The details of different modular are discussed as follows.

### 3.1. Image Enhancement

**Figure 7:**
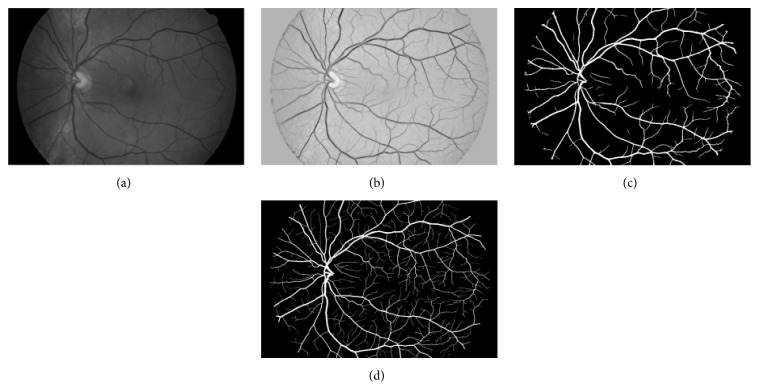
Segmentation of cake filter. (a) Green channel of image; (b) mixed real component; (c) segmentation result; (d) manual result.

In order to enhance segmentation effect, the first step is image preprocessing which aims to enhance the contrast of the image. As the cake filter can detect the details of lower contrast component, this work only uses simple image enhancement to reduce the amount of calculation. Through the observation of color retinal image, the green channel has the largest contrast which is shown in Figure 7(a). Its grayscale of most pixel is marked as [*a*, *b*]. The following is gray stretch, which is stretching the grayscale to the whole range marked as [0,255]

(7) $$\begin{array}{c} g\left(\;x,y\;\right)=\frac{f\left(x,y\right)-a}{b-a}\times 255, \end{array}$$

where *f* is the original image and *g* is the stretched image, *a* is the base level of gray value, and *b* is the top level.

### 3.2. Vessel Segmentation Based on Cake Filter and Fusion of Orientation Score

Figures 2 and 3 show that the real component of orientation score appears to be in a smaller gray scale after filtering retinal image, which is corresponding to the vessels and can be used to detect the vascular vessels. The imaginary part appears to be two regions, a lighter one and a darker one, corresponding to the boundary of vessels. In the current work, the real component is used to extract the retinal blood vessels. For cake filter, the value of *N* in Fig. is set to 32, which means there are 32 directions to detect the vessels. After getting the orientation scores from 32 directions, it needs a fusion of the real component and traversal of each pixel of the image. Combined with all the minimum of each real component that belongs to the corresponding direction, there comes the preliminary vessel network, shown as

(8) $$\begin{array}{c} g\left(\;i,j\;\right)=\min\left\{\;U_{f}1\left(\;i,j\;\right),U_{f}2\left(\;i,j\;\right),\ldots ,U_{f}n\left(\;i,j\;\right)\;\right\}, \end{array}$$

where *g* is the fused images and *U*_*f*_ is the real component of orientation score.

The result after fusion is shown in Figure 5. It can be seen that the vessels have a good enhancement, keeping many small vessels while extracting the main vascular with most noise filtered.

### 3.3. Vessel Network Extraction by Threshold

Though the difference on gray scale between the vessel and background is clear, the extraction has not fully finished after the fusion of real component; therefore, a self-adaptive threshold algorithm is used to extract vascular network.

First of all, after the fusion of real component, the image should be inverted to bring a high gray level on vessels while background appears to be a low gray level with a larger number. The threshold value is defined as *T* with an equation shown as

(9) $$\begin{array}{c} T=\min\left\{\;i\;|\;H\left(\;i\;\right)<h,\;\;i=g_{\min},g_{\min}+1,\ldots ,255\;\right\}, \end{array}$$

where *T* is the threshold value, *H*(*i*) is the number of pixels that belongs to the gray level *i*, *g*_min_ is the gray level which the most number of pixels belongs to, and *h* is the parameter made artificial. At last, *T* is used to binarize the image which is processed to fuse the real components and get the final result of vessel network.

## 4. Experiment Results and Analysis

To verify the proposed new vessel segmentation algorithm, a large number of experiments have been carried out to show its performance. Through analysis and comparison of experimental results, it is shown that when the value of *h* is set to 1500, it will achieve the best result of extraction of vascular network. The concrete results are shown in Figure 7, and Figure 7(c) comes to be the final segmentation.

From the results as shown in Figure 7, it can be seen that all main blood vessels have been split out. Also, most of the tiny blood vessels have the same results, which prove the effectiveness of the proposed algorithm. But comparing with experts segmentation results, there are still small blood vessels with low contrast that are not partition.

**Figure 8:**
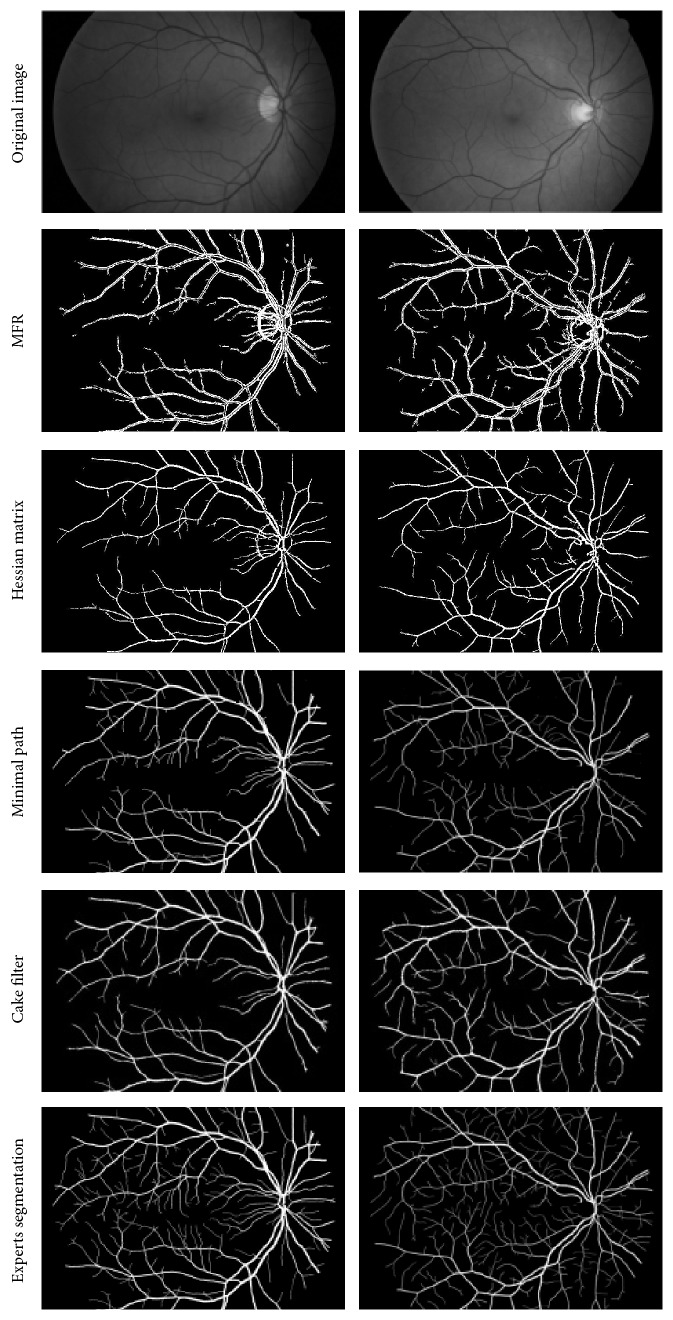
Comparison of segmentation methods.

Also, the comparisons with different algorithms and analysis have been carried out and the results are shown in Figure 8. Among the different algorithms, there are several classic algorithms and some are proposed recently. From top to bottom, there are the original image of retina, the segmentation results based on the matched filtering method [9], the extraction results based on the Hessian matrix method [14], the segmentation research based on minimal path method [21], the segmentation results based on cake filter, and the manual segmentation result from experts, as shown in Figure 8.

From Figure 8, it can be seen that the classical matched filtering method can segment the blood vessels, which have a large contrast. After segmentation, the vessel became thicker, and there are some which were empty; many of the small blood vessels are not segmented. It is obvious that the MFR is not ideal. For the method based on Hessian matrix, it can improve the result, but the final segmentation is also unsatisfactory on the details. About the algorithm proposed recently of minimal path, it can segment the most vessels with low contrast, and it is stronger than any of the above algorithms. But at the end of vessels, there are still some details at loss. The algorithm based on cake filter can get more details of vessel than others, and it proves that the proposed algorithm is better than other algorithms.

**Figure 9:**
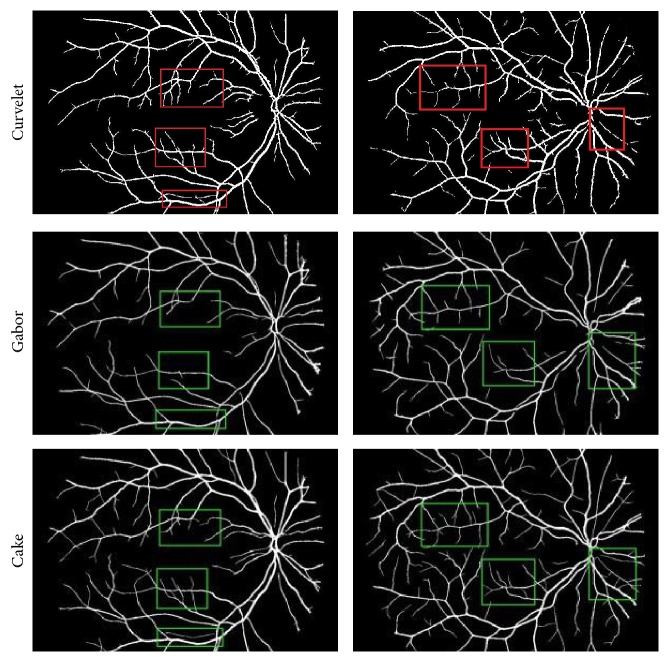
Segmentation with curvelet, Gabor, and cake.

Figure 9 shows the comparison about geometric tight frame (taking the curvelet, e.g.), Gabor, and cake filter. The three methods have been widely used for directional filtering, in which they all divide the frequency domain to get the directivity. Through the comparison of the three groups, it can be seen that although the three methods are efficient to get the capillaries the cake filter has more details and a better performance on the whole, while the Gabor filter fails in the details and the curvelet filter has problems of breaking links and noise.

**Figure 10:**
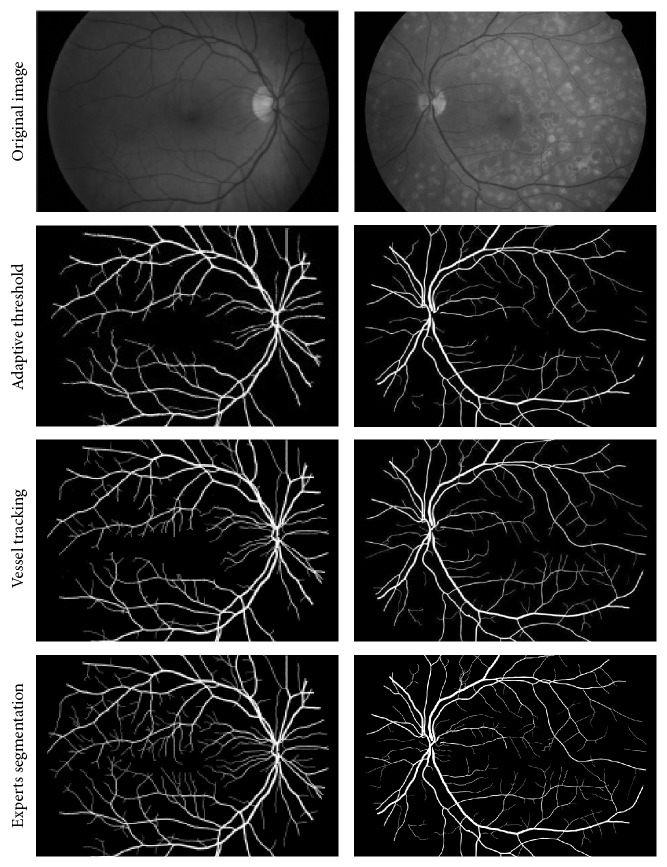
Comparison with threshold and vessel tracking.

In the final stage of algorithm, the method of adaptive threshold is used to finish the extraction. Compared with vessel tracking techniques, the threshold method has a higher operation efficiency, with a narrow loss in accuracy.  Figure 10 and Table 3 show the comparison in accuracy and stability between the adaptive threshold and the vessel tracking using both normal images and lesion images. It can be seen that although the threshold method has a less accuracy it performs better in operation time and has a more important role for the clinic application.

In order to further illustrate the segmentation results of the algorithm, we use (10) to calculate the average accuracy, sensitivity, and false positive rate. The result is shown in Table 2. Consider

(10) Ac=TN+BNNvp+Nuvp,TPR=TNNvp,FPR=FNNuvp,

where Ac is the average accuracy of retinal blood segmentation and TPR is the true positive rate, representing the fraction of pixels correctly detected as vessel pixels; FPR is the 1 − false positive rate, the fraction of pixels erroneously detected as vessel pixels; Nvp is the number of vessel pixels in the standard segmentation result; Nuvp is the number of background pixels in the standard segmentation result; TN is the true number where a pixel is identified as vessel in both the ground truth and segmented image; BN is the true background number where a pixel is identified as background in both the ground truth and segmented image; FN is the false number where a pixel is classified as nonvessel in both ground truth and segmented image.

At the same time, the segmentation results of the cake filter algorithm also are compared with other classic segmentation algorithms, and the results are shown in Table 4. From Table 4, the proposed algorithm has 0.962402 accuracy rate, which is superior to others, and the true positive rate (TPR) and the false positive rate (FPR) are also superior to others.

## 5. Conclusion

Starting with the characteristics of the retinal image and the advantages and disadvantages of the existing algorithm, the current work proposes a new algorithm for the retinal blood vessels segmentation based on cake filter. The algorithm first fuses the real component of orientation score using the cake filter and combines with the adaptive threshold value to get retinal vascular network. Then the algorithm adopts the image of STARE database to verify and analysis to show the performance. Experimental results prove the validation of this method on the extraction of retinal vascular network, especially for low contrast and small blood vessels, and the method has a better performance compared to the traditional segmentation algorithms on the average accuracy rate and true and false positive rate.

Although the algorithm of segmentation has been achieved with a certain performance, there is also a larger ascension space on the true positive rate and false positive rate of blood vessels segmentation, which will be studied in the future research.

## Figures and Tables

**Table 1:** Cake filter matched different *N* values.

*N*	Real component	Imaginary component	Fourier domain	Figure of Fourier
12	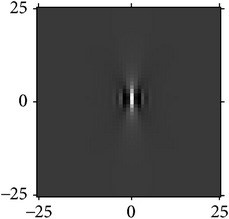	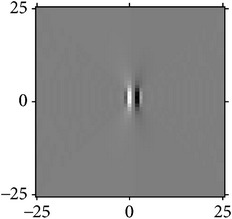	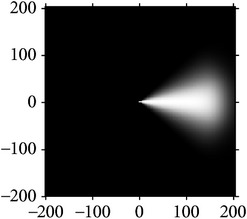	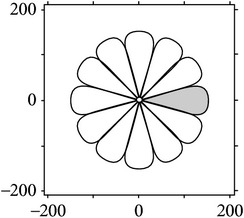

36	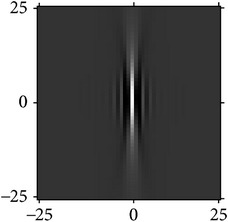	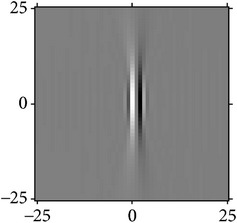	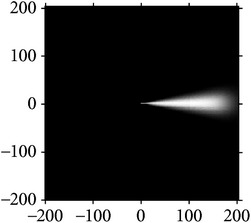	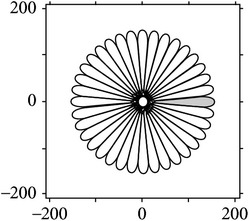

64	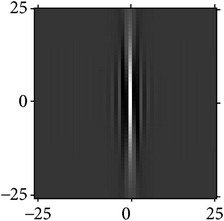	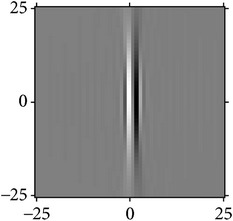	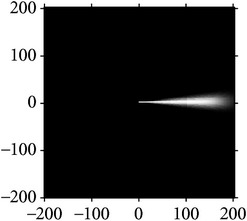	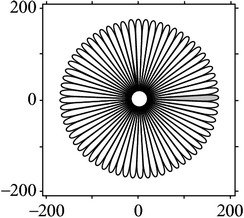

Gabor	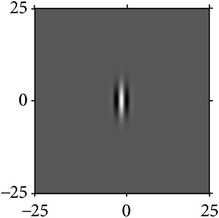	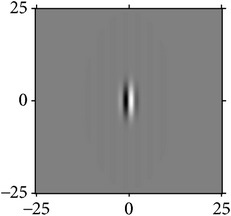	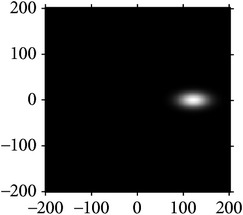	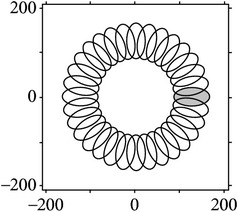

**Table 2:** Performance analysis of cake filter.

Image number	Average accuracy	True positive rate	1 − false positive rate
Test 1	0.95947	0.83486	0.033437
Test 2	0.96422	0.76743	0.044402
Test 3	0.96034	0.79549	0.032673
Test 4	0.96344	0.76064	0.041912
Test 5	0.96268	0.75466	0.042986
Test 6	0.96556	0.79759	0.042741
Test 7	0.96712	0.79237	0.041883
Test 8	0.97035	0.81147	0.040064
Test 9	0.95548	0.76761	0.036347
Test 10	0.96181	0.79721	0.035631

Average	0.962402	0.781224	0.038858

**Table 3:** Performance analysis of threshold and vessel extraction.

Method	Average accuracy	True positive rate	1 − false positive rate	Running time (s)
Threshold	0.96240	0.78122	0.03886	37.64
Vessel tracking	0.96853	0.79242	0.03619	483.05

**Table 4:** Comparison of segmentation result.

Algorithm	Average accuracy	True positive rate	1 − false positive rate
MFR	0.90439	0.60368	0.073911
Hessian	0.94116	0.63969	0.059122
Gabor	0.95413	0.69824	0.043661
Minimal path	0.95683	0.73367	0.041377
Cake	0.96242	0.78122	0.038858
